# Diiodido[4′-(4-pyrid­yl)-2,2′:6′,2′′-terpyridine-κ^3^ *N*,*N*′,*N*′′]copper(II)

**DOI:** 10.1107/S1600536810020635

**Published:** 2010-06-05

**Authors:** Feng-Tao Chen

**Affiliations:** a Department of Applied Chemistry, Zhejiang Sci-Tech University, Hangzhou 310018, People’s Republic of China and Key Laboratory of Advanced Textile Materials and Manufacturing Technology, Ministry of Education, Zhejiang Sci-Tech University, Hangzhou 310018, People’s Republic of China

## Abstract

The Cu^II^ atom in the title compound, [CuI_2_(C_20_H_14_N_4_)], has a distorted square-pyramidal coordination formed by the N atoms of the tridentate 4′-(4-pyrid­yl)-2,2′:6′2′′-terpyridine (pyterpy) ligand and two I atoms; one of the I atoms is in the apical position. In contrast to other known square-pyramidal diiodido- and dibromidocopper complexes of the pyterpy ligand in which metal–halogen distances are significantly different, in the title compound the apical and equatorial Cu—I bonds are almost identical [2.6141 (8) and 2.6025 (8) Å, respectively].

## Related literature

For related structures, see: Feng *et al.* (2006 ▶); Hou *et al.* (2004 ▶, 2005 ▶); Kutoglu *et al.* (1991 ▶); Shi *et al.* (2007 ▶); Zhang *et al.* (2007 ▶).
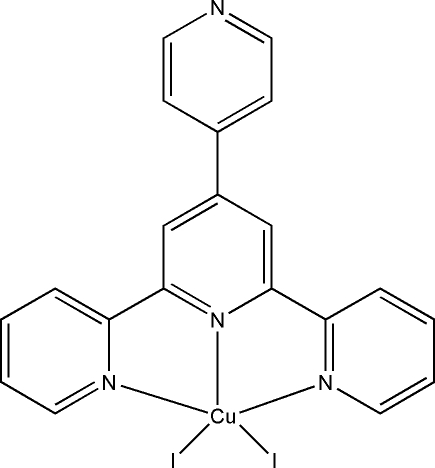


## Experimental

### 

#### Crystal data

[CuI_2_(C_20_H_14_N_4_)]
*M* *_r_* = 627.69
Monoclinic, 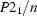

*a* = 11.9882 (8) Å
*b* = 14.642 (1) Å
*c* = 12.0291 (8) Å
β = 110.240 (1)°
*V* = 1981.1 (2) Å^3^
*Z* = 4
Mo *K*α radiation
μ = 4.23 mm^−1^
*T* = 294 K
0.25 × 0.23 × 0.18 mm

#### Data collection

Bruker SMART CCD diffractometer
Absorption correction: multi-scan (*SADABS*; Sheldrick, 1996 ▶) *T*_min_ = 0.361, *T*_max_ = 0.467
11694 measured reflections
3894 independent reflections
3670 reflections with *I* > 2σ(*I*)
*R*_int_ = 0.028

#### Refinement

*R*[*F* ^2^ > 2σ(*F* ^2^)] = 0.049
*wR*(*F* ^2^) = 0.091
*S* = 1.30
3894 reflections
244 parameters
H-atom parameters constrained
Δρ_max_ = 0.66 e Å^−3^
Δρ_min_ = −0.97 e Å^−3^

### 

Data collection: *SMART* (Bruker, 1998 ▶); cell refinement: *SAINT* (Bruker, 1998 ▶); data reduction: *SAINT*; program(s) used to solve structure: *SHELXS97* (Sheldrick, 2008 ▶); program(s) used to refine structure: *SHELXL97* (Sheldrick, 2008 ▶); molecular graphics: *SHELXTL* (Sheldrick, 2008 ▶); software used to prepare material for publication: *SHELXTL*.

## Supplementary Material

Crystal structure: contains datablocks I, global. DOI: 10.1107/S1600536810020635/ya2124sup1.cif

Structure factors: contains datablocks I. DOI: 10.1107/S1600536810020635/ya2124Isup2.hkl

Additional supplementary materials:  crystallographic information; 3D view; checkCIF report

## Figures and Tables

**Fig. 1.**
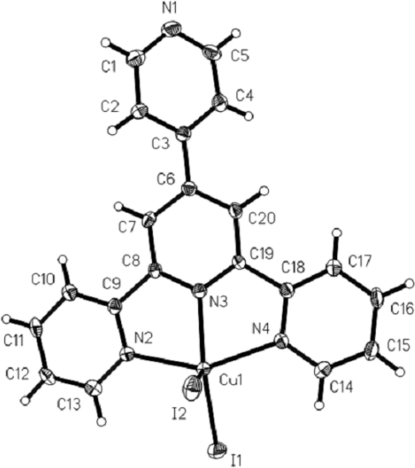
The asymmetric unit of the title compound showing 30% probability ellipsoids.

## supplementary crystallographic information

### Comment

Terpyridine and its derivatives have been recently receiving increasing attention not only because of their versatility as building blocks in supramolecular assemblies, but also due to the interesting electronic, photonic and magnetic properties of their transition metal complexes.

4'-(4-Pyridyl)-2,2':6'2''-terpyridine (pyterpy) belongs to this group of ligands and has been used to construct a great variety of structurally interesting entities, such as mononuclear complexes (Feng *et al.*, 2006; Hou *et al.*, 2004; Kutoglu *et al.*, 1991; Shi *et al.*, 2007), grid-type coordination polymers (Hou *et al., *2005), and self-catenated networks (Zhang *et al.*, 2007).

The structure of the title compound is shown in Fig. 1. The Cu1 atom has a distorted square-pyramidal coordination formed by the N2, N3 and N4 atoms of the pyterpy ligand and the I1 and I2 atoms. The I2 atom occupies the apical position, with bond angles of I2-Cu1-I1, I2-Cu1-N1, I2-Cu1-N2 and I2-Cu1-N3 being equal to 110.98 (3)°, 102.6 (1)°, 107.9 (1)° and 97.3 (1)° respectively, and the widest bond angles in the coordination sphere of the Cu1 atom being I1-Cu1-N3 [141.1 (1)°] and N2-Cu1-N4 [146.4 (2)°].

Rather unexpectedly, in contrast with other diiodo- and dibromo-copper complexes of 2,2':6'2''-terpyridine (terpy)  with square-pyramidal coordination (Hou *et al.*,  2004; Feng *et al.*, 2006), where significant difference between the apical and equatorial metal-halogen bonds was observed, in the title compound the Cu1-I1 and Cu1-I2 bonds are almost identical [2.6025 (8) Å and 2.6141 (8) Å  respectively]. It is true, however, that the wide angles in the copper coordination sphere (I1-Cu1-N3 and N2-Cu1-N4) are significantly narrower in the title compound than in other terpy complexes with square-pyramidal configuration (see references above), which puts this compound much farther on the transition path to trigonal bipyramid than the mentioned above literature complexes.

The Cu1-N3 bond with the N atom of the central ring of the pyterpy ligand [2.104 (4) Å] is noticeably shorter, than the Cu1-N2 and Cu1-N4 bonds [2.206 (5) Å and 2.199 (5) Å] involving the  flanking pyridine rings of the pyterpy ligand. This pattern in the Cu-N bonds, is quite typical for the terpy complexes (see Hou *et al.*,  2004; Feng *et al.*,  2006; Kutoglu *et al.*,  1991).

### Experimental

The mixture of CuI (0.0190 g, 0.1 mmol), 4'-(4-pyridyl)-2,2':6'2''-terpyridine (pyterpy) (0.0155 g, 0.05 mmol), saturated KI solution (3 ml) and water (6 ml) were placed and sealed in a 10 ml Teflon-lined stainless steel reactor and heated to 140 °C for 72 h, then cooled down to room temperature at a rate of 2°C/20 min. Single crystals suitable for X-ray diffraction were obtained in the form of black bars in *ca* 40% yield.

### Refinement

H atoms were positioned geometrically and refined using a riding model with C—H = 0.93 Å(aromatic) and *U*_ĩso_(H) = 1.2*U*_eq_(C)

### Crystal data

**Table:**

[CuI_2_(C_20_H_14_N_4_)]	*F*(000) = 1188
*M**_r_* = 627.69	*D*_x_ = 2.105 Mg m^−^^3^
Monoclinic, *P*2_1_/*n*	Mo *K*α radiation, λ = 0.71073 Å
Hall symbol: -P 2yn	Cell parameters from 1523 reflections
*a* = 11.9882 (8) Å	θ = 1.9–20.3°
*b* = 14.642 (1) Å	µ = 4.23 mm^−^^1^
*c* = 12.0291 (8) Å	*T* = 294 K
β = 110.240 (1)°	Block, black
*V* = 1981.1 (2) Å^3^	0.25 × 0.23 × 0.18 mm
*Z* = 4	

### Data collection

**Table:**

Bruker SMART CCD diffractometer	3894 independent reflections
Radiation source: fine-focus sealed tube	3670 reflections with *I* > 2σ(*I*)
graphite	*R*_int_ = 0.028
phi and ω scans	θ_max_ = 26.0°, θ_min_ = 2.1°
Absorption correction: multi-scan (*SADABS*; Sheldrick, 1996)	*h* = −14→13
*T*_min_ = 0.361, *T*_max_ = 0.467	*k* = −18→17
11694 measured reflections	*l* = −8→14

### Refinement

**Table:**

Refinement on *F*^2^	Primary atom site location: structure-invariant direct methods
Least-squares matrix: full	Secondary atom site location: difference Fourier map
*R*[*F*^2^ > 2σ(*F*^2^)] = 0.049	Hydrogen site location: inferred from neighbouring sites
*wR*(*F*^2^) = 0.091	H-atom parameters constrained
*S* = 1.30	*w* = 1/[σ^2^(*F*_o_^2^) + (0.0189*P*)^2^ + 6.8023*P*] where *P* = (*F*_o_^2^ + 2*F*_c_^2^)/3
3894 reflections	(Δ/σ)_max_ = 0.001
244 parameters	Δρ_max_ = 0.66 e Å^−^^3^
0 restraints	Δρ_min_ = −0.97 e Å^−^^3^

### Special details

**Table:**

Geometry. All esds (except the esd in the dihedral angle between two l.s. planes) are estimated using the full covariance matrix. The cell esds are taken into account individually in the estimation of esds in distances, angles and torsion angles; correlations between esds in cell parameters are only used when they are defined by crystal symmetry. An approximate (isotropic) treatment of cell esds is used for estimating esds involving l.s. planes.
Refinement. Refinement of *F*^2^ against ALL reflections. The weighted *R*-factor wR and goodness of fit *S* are based on *F*^2^, conventional *R*-factors *R* are based on *F*, with *F* set to zero for negative *F*^2^. The threshold expression of *F*^2^ > σ(*F*^2^) is used only for calculating *R*-factors(gt) etc. and is not relevant to the choice of reflections for refinement. *R*-factors based on *F*^2^ are statistically about twice as large as those based on *F*, and *R*- factors based on ALL data will be even larger.

### Fractional atomic coordinates and isotropic or equivalent isotropic displacement parameters (Å^2^)

**Table:**

	*x*	*y*	*z*	*U*_iso_*/*U*_eq_	
Cu1	0.73294 (6)	0.14757 (4)	0.92788 (6)	0.02734 (17)	
C1	1.1049 (6)	−0.1207 (5)	0.5460 (6)	0.0409 (15)	
H1	1.0765	−0.1717	0.4986	0.049*	
C2	1.0403 (5)	−0.0897 (4)	0.6143 (5)	0.0346 (14)	
H2	0.9724	−0.1206	0.6142	0.042*	
C3	1.0786 (5)	−0.0118 (4)	0.6828 (5)	0.0288 (12)	
C4	1.1830 (5)	0.0295 (4)	0.6800 (5)	0.0343 (13)	
H4	1.2126	0.0818	0.7241	0.041*	
C5	1.2409 (6)	−0.0091 (4)	0.6106 (6)	0.0411 (16)	
H5	1.3108	0.0187	0.6108	0.049*	
C6	1.0106 (5)	0.0250 (4)	0.7539 (5)	0.0278 (12)	
C7	0.9499 (5)	−0.0329 (4)	0.8056 (5)	0.0304 (13)	
H7	0.9566	−0.0959	0.8006	0.036*	
C8	0.8799 (5)	0.0042 (4)	0.8644 (5)	0.0253 (11)	
C9	0.8078 (5)	−0.0505 (4)	0.9195 (5)	0.0279 (12)	
C10	0.8152 (5)	−0.1438 (4)	0.9284 (5)	0.0347 (13)	
H10	0.8651	−0.1760	0.8981	0.042*	
C11	0.7474 (6)	−0.1892 (4)	0.9830 (6)	0.0421 (16)	
H11	0.7519	−0.2523	0.9916	0.050*	
C12	0.6724 (6)	−0.1390 (4)	1.0249 (6)	0.0442 (16)	
H12	0.6249	−0.1677	1.0612	0.053*	
C13	0.6698 (6)	−0.0452 (4)	1.0115 (6)	0.0404 (15)	
H13	0.6197	−0.0115	1.0400	0.048*	
C14	0.8092 (6)	0.3530 (4)	0.9194 (6)	0.0383 (14)	
H14	0.7541	0.3645	0.9563	0.046*	
C15	0.8637 (6)	0.4263 (4)	0.8878 (6)	0.0391 (15)	
H15	0.8446	0.4858	0.9016	0.047*	
C16	0.9473 (6)	0.4096 (4)	0.8354 (6)	0.0417 (16)	
H16	0.9863	0.4577	0.8140	0.050*	
C17	0.9724 (6)	0.3199 (4)	0.8149 (6)	0.0384 (15)	
H17	1.0288	0.3069	0.7801	0.046*	
C18	0.9120 (5)	0.2504 (4)	0.8473 (5)	0.0271 (12)	
C19	0.9295 (5)	0.1515 (4)	0.8282 (5)	0.0259 (12)	
C20	1.0022 (5)	0.1191 (4)	0.7688 (5)	0.0298 (12)	
H20	1.0448	0.1595	0.7392	0.036*	
I1	0.69282 (4)	0.20067 (3)	1.11708 (4)	0.04474 (14)	
I2	0.54357 (4)	0.16793 (3)	0.73907 (4)	0.04683 (14)	
N1	1.2047 (5)	−0.0826 (4)	0.5436 (5)	0.0455 (14)	
N2	0.7357 (4)	−0.0010 (3)	0.9595 (4)	0.0310 (11)	
N3	0.8694 (4)	0.0951 (3)	0.8736 (4)	0.0267 (10)	
N4	0.8313 (4)	0.2670 (3)	0.8997 (4)	0.0298 (11)	

### Atomic displacement parameters (Å^2^)

**Table:**

	*U*^11^	*U*^22^	*U*^33^	*U*^12^	*U*^13^	*U*^23^
Cu1	0.0286 (4)	0.0248 (3)	0.0331 (4)	0.0006 (3)	0.0162 (3)	−0.0012 (3)
C1	0.046 (4)	0.039 (4)	0.040 (4)	0.006 (3)	0.018 (3)	−0.001 (3)
C2	0.037 (3)	0.030 (3)	0.041 (4)	0.003 (2)	0.019 (3)	−0.003 (3)
C3	0.031 (3)	0.026 (3)	0.031 (3)	0.007 (2)	0.012 (3)	0.005 (2)
C4	0.036 (3)	0.031 (3)	0.034 (3)	0.002 (3)	0.011 (3)	0.007 (3)
C5	0.038 (3)	0.043 (4)	0.052 (4)	0.010 (3)	0.027 (3)	0.013 (3)
C6	0.027 (3)	0.029 (3)	0.029 (3)	0.001 (2)	0.011 (2)	0.001 (2)
C7	0.038 (3)	0.020 (3)	0.032 (3)	0.003 (2)	0.011 (3)	−0.001 (2)
C8	0.026 (3)	0.023 (3)	0.025 (3)	−0.001 (2)	0.006 (2)	0.003 (2)
C9	0.025 (3)	0.027 (3)	0.029 (3)	−0.003 (2)	0.005 (2)	−0.002 (2)
C10	0.036 (3)	0.032 (3)	0.038 (3)	−0.002 (2)	0.016 (3)	0.002 (3)
C11	0.050 (4)	0.026 (3)	0.054 (4)	−0.011 (3)	0.022 (3)	0.006 (3)
C12	0.045 (4)	0.044 (4)	0.054 (4)	−0.014 (3)	0.030 (3)	0.003 (3)
C13	0.038 (3)	0.040 (4)	0.048 (4)	−0.004 (3)	0.022 (3)	−0.003 (3)
C14	0.043 (4)	0.032 (3)	0.044 (4)	0.005 (3)	0.019 (3)	−0.003 (3)
C15	0.043 (4)	0.023 (3)	0.049 (4)	−0.001 (3)	0.014 (3)	−0.001 (3)
C16	0.043 (4)	0.024 (3)	0.060 (4)	−0.007 (3)	0.019 (3)	0.006 (3)
C17	0.041 (4)	0.029 (3)	0.051 (4)	0.000 (3)	0.023 (3)	0.002 (3)
C18	0.024 (3)	0.025 (3)	0.031 (3)	0.001 (2)	0.008 (2)	0.004 (2)
C19	0.030 (3)	0.023 (3)	0.028 (3)	−0.002 (2)	0.014 (2)	0.003 (2)
C20	0.035 (3)	0.025 (3)	0.031 (3)	−0.005 (2)	0.014 (3)	0.003 (2)
I1	0.0565 (3)	0.0431 (3)	0.0441 (3)	0.0026 (2)	0.0296 (2)	−0.0038 (2)
I2	0.0424 (3)	0.0349 (2)	0.0504 (3)	−0.00135 (18)	−0.0003 (2)	0.0007 (2)
N1	0.054 (4)	0.041 (3)	0.052 (4)	0.013 (3)	0.032 (3)	0.001 (3)
N2	0.033 (3)	0.027 (2)	0.038 (3)	0.001 (2)	0.019 (2)	0.000 (2)
N3	0.029 (2)	0.025 (2)	0.027 (2)	0.0012 (19)	0.010 (2)	0.0025 (19)
N4	0.028 (2)	0.027 (2)	0.036 (3)	−0.0025 (19)	0.013 (2)	0.000 (2)

### Geometric parameters (Å, °)

**Table:**

Cu1—N3	2.104 (4)	C9—C10	1.370 (8)
Cu1—N4	2.199 (5)	C10—C11	1.380 (8)
Cu1—N2	2.206 (5)	C10—H10	0.9300
Cu1—I1	2.6025 (8)	C11—C12	1.383 (9)
Cu1—I2	2.6141 (8)	C11—H11	0.9300
C1—N1	1.330 (8)	C12—C13	1.381 (9)
C1—C2	1.386 (8)	C12—H12	0.9300
C1—H1	0.9300	C13—N2	1.333 (7)
C2—C3	1.389 (8)	C13—H13	0.9300
C2—H2	0.9300	C14—N4	1.325 (7)
C3—C4	1.401 (8)	C14—C15	1.376 (9)
C3—C6	1.473 (7)	C14—H14	0.9300
C4—C5	1.379 (8)	C15—C16	1.378 (9)
C4—H4	0.9300	C15—H15	0.9300
C5—N1	1.324 (9)	C16—C17	1.388 (8)
C5—H5	0.9300	C16—H16	0.9300
C6—C7	1.396 (8)	C17—C18	1.381 (8)
C6—C20	1.397 (8)	C17—H17	0.9300
C7—C8	1.382 (8)	C18—N4	1.347 (7)
C7—H7	0.9300	C18—C19	1.493 (7)
C8—N3	1.345 (7)	C19—N3	1.331 (6)
C8—C9	1.492 (7)	C19—C20	1.387 (8)
C9—N2	1.340 (7)	C20—H20	0.9300
			
N3—Cu1—N4	74.14 (17)	C10—C11—C12	118.8 (6)
N3—Cu1—N2	74.17 (17)	C10—C11—H11	120.6
N4—Cu1—N2	146.45 (17)	C12—C11—H11	120.6
N3—Cu1—I1	141.15 (13)	C13—C12—C11	118.5 (6)
N4—Cu1—I1	99.68 (13)	C13—C12—H12	120.7
N2—Cu1—I1	98.03 (13)	C11—C12—H12	120.7
N3—Cu1—I2	107.86 (13)	N2—C13—C12	123.0 (6)
N4—Cu1—I2	97.26 (13)	N2—C13—H13	118.5
N2—Cu1—I2	102.65 (13)	C12—C13—H13	118.5
I1—Cu1—I2	110.98 (3)	N4—C14—C15	123.3 (6)
N1—C1—C2	124.5 (6)	N4—C14—H14	118.4
N1—C1—H1	117.7	C15—C14—H14	118.4
C2—C1—H1	117.7	C14—C15—C16	118.5 (6)
C1—C2—C3	118.9 (6)	C14—C15—H15	120.7
C1—C2—H2	120.6	C16—C15—H15	120.7
C3—C2—H2	120.6	C15—C16—C17	119.1 (6)
C2—C3—C4	117.0 (5)	C15—C16—H16	120.5
C2—C3—C6	120.7 (5)	C17—C16—H16	120.5
C4—C3—C6	122.3 (5)	C18—C17—C16	118.7 (6)
C5—C4—C3	118.7 (6)	C18—C17—H17	120.7
C5—C4—H4	120.7	C16—C17—H17	120.7
C3—C4—H4	120.7	N4—C18—C17	122.1 (5)
N1—C5—C4	125.0 (6)	N4—C18—C19	114.1 (5)
N1—C5—H5	117.5	C17—C18—C19	123.8 (5)
C4—C5—H5	117.5	N3—C19—C20	121.7 (5)
C7—C6—C20	118.0 (5)	N3—C19—C18	114.4 (5)
C7—C6—C3	121.0 (5)	C20—C19—C18	123.9 (5)
C20—C6—C3	121.0 (5)	C19—C20—C6	119.3 (5)
C8—C7—C6	119.4 (5)	C19—C20—H20	120.4
C8—C7—H7	120.3	C6—C20—H20	120.4
C6—C7—H7	120.3	C5—N1—C1	115.9 (5)
N3—C8—C7	121.5 (5)	C13—N2—C9	117.8 (5)
N3—C8—C9	114.2 (5)	C13—N2—Cu1	125.8 (4)
C7—C8—C9	124.3 (5)	C9—N2—Cu1	116.4 (4)
N2—C9—C10	123.0 (5)	C19—N3—C8	120.0 (5)
N2—C9—C8	114.4 (5)	C19—N3—Cu1	119.4 (3)
C10—C9—C8	122.6 (5)	C8—N3—Cu1	119.5 (4)
C9—C10—C11	119.0 (6)	C14—N4—C18	118.4 (5)
C9—C10—H10	120.5	C14—N4—Cu1	125.3 (4)
C11—C10—H10	120.5	C18—N4—Cu1	116.0 (4)
